# Novel Cooperative Automatic Modulation Classification Using Vectorized Soft Decision Fusion for Wireless Sensor Networks

**DOI:** 10.3390/s22051797

**Published:** 2022-02-24

**Authors:** Xiao Yan, Yan Zhang, Xiaoxue Rao, Qian Wang, Hsiao-Chun Wu, Yiyan Wu

**Affiliations:** 1 School of Aeronautics and Astronautics, University of Electronic Science and Technology of China, Chengdu 611731, China; yanxiao@uestc.edu.cn (X.Y.); zhangryan@std.uestc.edu.cn (Y.Z.); raoxiaoxue@std.uestc.edu.cn (X.R.); 2 School of Electrical Engineering and Computer Science, Louisiana State University, Baton Rouge, LA 70803, USA; wu@ece.lsu.edu; 3 Communications Research Centre, Ottawa, ON K2H 8S2, Canada; yiyan.wu@ieee.org

**Keywords:** cooperative automatic modulation classification (CAMC), vectorized decision metrics, soft-decision-level fusion, graph-based automatic modulation classification, Hamming distance sequence

## Abstract

Cooperative automatic modulation classification (CAMC) using a swarm of sensors is intriguing nowadays as it would be much more robust than the conventional single-sensing-node automatic modulation classification (AMC) method. We propose a novel robust CAMC approach using vectorized soft decision fusion in this work. In each sensing node, the local Hamming distances between the graph features acquired from the unknown target signal and the training modulation candidate signals are calculated and transmitted to the fusion center (FC). Then, the global CAMC decision is made by the indirect vote which is translated from each sensing node’s Hamming-distance sequence. The simulation results demonstrate that, when the signal-to-noise ratio (SNR) was given by η ≥ 0dB, our proposed new CAMC scheme’s correct classification probability $P_{\mathrm{cc}}$ could reach up close to 100%. On the other hand, our proposed new CAMC scheme could significantly outperform the single-node graph-based AMC technique and the existing decision-level CAMC method in terms of recognition accuracy, especially in the low-SNR regime.

## 1. Introduction

Automatic modulation classification (AMC) mechanisms can enable the frontend of cognitive ratio technology by blindly identifying the modulation scheme of the transmitted signal. AMC techniques are also very useful in military and civilian applications such as cognitive radio, adaptive modulation, dynamic spectrum access, surveillance and electronic warfare [1,2,3,4,5,6]. Generally, conventional AMC approaches can be split into two major categories, (i) the maximum-likelihood-based (ML) approach and (ii) the feature-recognition-based (FR) approach [7]. In practice, the FR methods are more popular than the ML methods, as the likelihood function of the observed signal data can often be complex and impossible to formulate precisely. On the other hand, the FR approach usually involves two key steps, namely, *feature extraction* and *modulation classification*. Commonly adopted features include wavelet-related features, cyclic spectrum, high-order statistics, etc. [8,9,10]. Furthermore, the majority of AMC research works in the literature is focused on the single-sensing-node paradigm, which is quite susceptible to bad channel conditions and/or high noise levels [11].

In recent years, wireless sensor networks (WSNs) have been emerging as solutions to many practical applications. Spatially distributed cooperative sensing nodes can infer more reliable statistical information than any individual sensing node, leading to a much more robust AMC performance [12,13]. A cooperative AMC method, though leading to a higher AMC accuracy than the single-sensing-node counterpart, was still rather sensitive to individual sensing nodes’ errors [14]. A reliable cooperative automatic modulation classification (CAMC) approach should facilitate a fusion center (FC), which fuses local information acquired and/or produced by individual sensing nodes according to [15]. Such fusion mechanisms can be implemented at the data, feature and decision levels. The raw signal data received by each sensing node is directly transmitted to the FC in a data-level fusion mechanism. Although the minimum processing burden is required for each sensing node, the data-level fusion mechanism would require a large transmission overhead from each sensing node to the FC. In a feature-level fusion mechanism, each sensing node independently extracts features from the received signal data and then transmits the extracted features to the FC, which requires all sensing nodes to be highly synchronized with each other for making the global AMC decision. The decision-level fusion mechanisms can be further split into two categories, namely, (i) *optimal hard decision fusion* (OHDF) mechanisms [16,17,18,19] and (ii) *soft decision fusion* (SDF) mechanisms [20]. In an OHDF mechanism, each sensing node makes a local decision based on its extracted features and then such a local decision is transmitted to the FC for making the global decision. In an SDF mechanism, each sensing node extracts features, converts the extracted features to a decision metric and transmits its decision metric to the FC; ultimately, the FC fuses the received decision metrics from all sensing nodes and makes the global AMC decision. Obviously, decision-level fusion mechanisms greatly reduce both the transmission overhead from each sensing node to the FC and the computational burden of the FC. Meanwhile, to combat the drawback whereby the OHDF mechanisms would often suffer from potential local-decision errors, we focus on the SDF approach in this work.

In this paper, we propose a new robust CAMC method based on the vectorized soft decision fusion (VSDF) mechanism (a new SDF scheme). In our proposed new CAMC approach, to identify the modulation type of an unknown target signal, each sensing node employs our graph-based AMC method, previously proposed in [21,22], to produce a decision-metric sequence, namely, the Hamming-distance sequence between the graph features acquired from the received signal data and all candidate modulations, and then transmit the decision-metric sequence to the FC. Finally, the FC applies our proposed new vectorized soft decision fusion mechanism to make the global AMC decision. The Monte Carlo simulation results and a linear discriminant analysis (LDA) showed that, in comparison with our CAMC method, recently proposed in [23], our proposed new CAMC method using the vectorized soft decision fusion mechanism is much more robust in terms of recognition accuracy, especially for low-signal-to-noise-ratio (SNR) conditions. The main contributions of this work are summarized as follows:

- In this work, a new CAMC framework is proposed; it outperformed the conventional single-node AMC approach, especially when individual channel conditions vary significantly.
- A novel vectorized soft decision fusion strategy using the *voting mechanism* based on the “perturbed” local normalized Hamming-distance sequences at the FC was theoretically derived, which can avoid potential local-decision errors arising from the OHDF mechanisms.
- By integrating the local graph-based AMC scheme at each individual sensing node and the new vectorized soft decision fusion strategy at the FC, we designed a new decision-level CAMC approach for distributed (decentralized) WSNs. Monte Carlo simulations demonstrated its superiority to the existing CAMC approach.

The rest of this paper is organized as follows: Section 2 introduces the CAMC system model in a WSN. The details of our proposed new CAMC scheme are discussed in Section 3. Monte Carlo simulation results and the associated LDA are presented in Section 4. Conclusion are finally drawn in Section 5.

## 2. System Model

**Figure 1:**
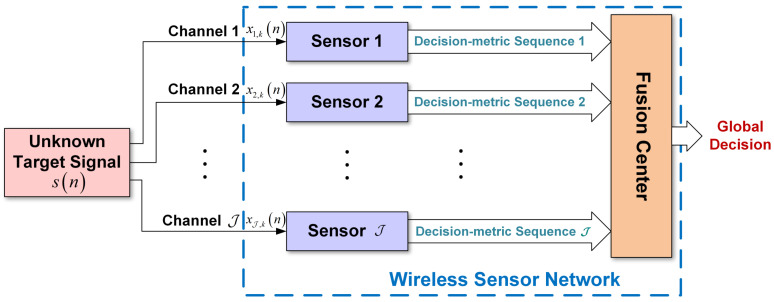
The system model of our proposed new CAMC scheme based on the vectorized soft decision fusion rule for wireless sensor networks.

A distributed wireless sensor network composed of J sensing devices (nodes) with an FC is facilitated to continuously identify the modulation type of an unknown target signal, which turns out to be a discrete-time sequence s(n), n=1, 2, *…*, *N* within an arbitrary sensing interval after sampling. Let us assume that a modulation candidate set $M\overset{\mathrm{def}}{=}\left\{M_{1},M_{2},\cdots ,M_{M}\right\}$ is pre-specified, where $M_{m}$ represents the *m*th modulation type, for m=1, 2, *…*, *M*. During the *k*th sensing interval, the unknown target signal $s_{k}\left(n\right)$ with modulation $M_{m}\in M$ is sensed by all J sensing nodes and the discrete-time received-signal sequence $x_{j,k}\left(n\right)$ at the *j*th sensing device, for j=1, 2, *…*, J is given by

(1) $$x_{j,k}\left(n\right)=h_{j,k}\left(n\right)⊗s_{k}\left(n\right)+w_{j,k}\left(n\right),\;\;n=1,2,\ldots ,N,$$

where $h_{j,k}\left(n\right)$ denotes the discrete-time finite-impulse-response (FIR) channel filter associated with the multipath channel corresponding to the *j*th sensing node; “⊗” denotes linear convolution; $w_{j,k}\left(n\right)$ denotes the additive white Gaussian noise (AWGN) sequence with zero mean and variance $\sigma_{j,k}^{2}$ appearing in the *k*th sensing interval. According to the system model illustrated by Figure 1, each sensing node independently extracts the modulation features of the unknown target signal $s_{k}\left(n\right)$ based on its received signal sequence $x_{j,k}\left(n\right)$ within the *k*th sensing interval. The local modulation features are formulated as the decision-metric sequences and are then transmitted to the fusion center of the WSN. The global decision of the modulation scheme $M_{m}\in M$ of the unknown target signal $s_{k}\left(n\right)$ is eventually made by the FC based on these aggregated local decision-metric sequences.

## 3. The Proposed Novel Cooperative AMC Approach

In accordance with the system model depicted by Figure 1, our proposed cooperative AMC approach using vectorized soft decision fusion is introduced here. By use of our graph-based AMC approach, proposed in [21], the collection of local soft decision metrics of a sensing node, each of which is the Hamming distance between the graph features extracted from the received signal and those from the training signal of a particular candidate modulation (refer to Section 3.1 below for details), can be produced and then transmitted to the FC. Such local soft decision metrics sent by all sensing nodes are collected by the FC to generate the weighted votes for all candidates in the modulation candidate set $M\overset{\mathrm{def}}{=}{\left\{M_{m}\right\}}_{m=1}^{M}$ such that the global decision can be made thereupon.

### 3.1. Local Graph-Based AMC Scheme

During the *k*th sensing interval, an *N*-sample signal sequence $x_{j,k}\left(n\right)$ is received by the *j*th sensing device. Our proposed graph-based AMC method in [21] is employed by all sensing nodes to produce local soft decision metrics. For the *j*th sensing device in the *k*th sensing interval, the corresponding cyclic spectrum (CS) $S_{x_{j,k}}^{\varepsilon }\left(f\right)$ of the received signal $x_{j,k}\left(n\right)$ is first estimated using a time-smooth algorithm called the FFT (fast Fourier transform) accumulation method (FAM) [21], which involves 2N+1 cyclic frequencies $\varepsilon =\varepsilon_{d}$, for d=−N, −N+1, *…*, *N*, according to [21]. Only a quadrant of ${\bar{S}}_{x_{j,k}}^{\varepsilon }\left(f\right)$ (normalized and quantized $S_{x_{j,k}}^{\varepsilon }\left(f\right)$) needs to be converted to N+1 graphs (one for each focused cyclic frequency $\varepsilon_{d}$) $G_{\varepsilon_{d}}\overset{\mathrm{def}}{=}\left(V_{\varepsilon_{d}},E_{\varepsilon_{d}}\right)$, for d=0, 1, *…*, *N*, according to the graph-mapping mechanism presented in [21]. As manifested in [21], from the noise-free training signal of the modulation $M_{m}\in M$, a set of graphs can be constructed from its CS as given by

(2) $$G_{m}\overset{\mathrm{def}}{=}\left\{G_{\varepsilon_{0}}^{m},G_{\varepsilon_{1}}^{m},\cdots ,G_{\varepsilon_{N}}^{m}\right\},\;\;m=1,2,\ldots ,M,$$

where $G_{\varepsilon_{d}}^{m}\overset{\mathrm{def}}{=}\left(V_{\varepsilon_{d}}^{m},E_{\varepsilon_{d}}^{m}\right)$ and the set of the corresponding adjacency matrices is given by

(3) $$A_{m}\overset{\mathrm{def}}{=}\left\{A_{\varepsilon_{0}}^{m},A_{\varepsilon_{1}}^{m},\cdots ,A_{\varepsilon_{N}}^{m}\right\},\;\;m=1,2,\ldots ,M,$$

where $A_{\varepsilon_{d}}^{m}$ is the adjacency matrix of $G_{\varepsilon_{d}}^{m}$ and more relevant details can be found in [23]. Then, one can produce the modulation feature sequence $I_{m}^{\mathrm{training}}$ for the *m*th modulation candidate $M_{m}\in M$ from $A_{m}$, m=1, 2, *…*, *M* by use of the Kullback–Leibler divergence of the dominant entries in the adjacency matrices in $A_{m}$. Furthermore, for all modulation candidates in M, a set of modulation feature sequences $I^{\mathrm{training}}\overset{\mathrm{def}}{=}\left\{I_{1}^{\mathrm{training}},I_{2}^{\mathrm{training}},\cdots ,I_{M}^{\mathrm{training}}\right\}$ can be formed. It should be pointed out that $I^{\mathrm{training}}$ remains unchanged across all sensing intervals and thus can be constructed and stored at all sensing nodes in advance.

During the test stage in the *k*th sensing interval, a set of graphs ${\tilde{G}}_{k,j}$ can also be constructed from the corresponding CS at the *j*th sensing node using the above-stated approach. The modulation feature sequences for the test signal can thus be formed as $I_{m,k,j}^{\mathrm{test}}$, m=1, 2, *…*, *M* from the corresponding adjacency-matrix set ${\tilde{A}}_{k,j}$ using the aforementioned procedure for producing the training modulation feature sequences. Note that both $I_{m,k,j}^{\mathrm{test}}$ and $I_{m}^{\mathrm{training}}$, m=1, 2, *…*, *M* have the same sequence length *L*, while pertinent details can also be found in [22]. Once the modulation feature sequences $I_{m,k,j}^{\mathrm{test}}$, m=1, 2, *…*, *M* for the test signal data are built by the *j*th sensing node in the *k*th sensing interval, the “*normalized Hamming distance*” (NHD) ${\bar{H}}_{m,j,k}$ between the feature sequence produced from the *m*th modulation candidate during training and that produced from the test signal can be calculated by

(4) $${\bar{H}}_{m,j,k}\overset{\mathrm{def}}{=}\frac{H\left(I_{m}^{\mathrm{training}},I_{m,k,j}^{\mathrm{test}}\right)}{L},$$

where $H\left(I_{m}^{\mathrm{training}},I_{m,k,j}^{\mathrm{test}}\right)$ denotes the Hamming distance between $I_{m}^{\mathrm{training}}$ and $I_{m,k,j}^{\mathrm{test}}$. For all modulation candidates in M, a set of NHDs, namely, $H_{j,k}=\left\{{\bar{H}}_{1,j,k},{\bar{H}}_{2,j,k},\cdots ,{\bar{H}}_{M,j,k}\right\}$, can be formed at the *j*th sensing device in the *k*th sensing interval and transmitted to the FC for finally reaching the global CAMC decision.

### 3.2. New Vectorized Feature Fusion Rule

Since the target source emits signals to spatially distributed sensing devices through different transmission paths, which lead to different channel conditions for different sensing nodes, the local AMC accuracies across individual sensing nodes are often very different. Such erroneous local decisions would negatively influence the global decision made by the FC. To combat this drawback, we propose a *novel vectorized soft decision fusion strategy*, which may mitigate the negative effect of poor local channel conditions.

In the *k*th sensing interval, all local NHD sequences $H_{j,k}$, j=1, 2, *…*, J, which are built by the individual sensing nodes using the graph-based AMC approach discussed in Section 3.1, are transmitted to the FC. At the FC, a small flooring constant ζ is introduced to those zero-valued NHDs in $H_{j,k}$, j=1, 2, *…*, J, such that $H_{j,k}$ is converted to ${\hat{H}}_{j,k}\overset{\mathrm{def}}{=}\left\{{\hat{H}}_{1,j,k},{\hat{H}}_{2,j,k},\cdots ,{\hat{H}}_{M,j,k}\right\}$, which is the “perturbed” local NHD sequence resulting from the *j*th sensing node in the *k*th sensing interval, where

(5) $${\hat{H}}_{m,j,k}\overset{\mathrm{def}}{=}\left\{\begin{array}{cc} {\bar{H}}_{m,j,k},\;\; & \mathrm{if}\;{\bar{H}}_{m,j,k}\neq 0,\\ \zeta ,\;\; & \mathrm{if}\;{\bar{H}}_{m,j,k}=0, \end{array}\right.$$

where ζ is the preset flooring constant. According to our heuristic experience, setting ζ to be less than or equal to $10^{-5}$ can lead to promising performance. Hence, ζ is fixed to be $10^{-5}$ here. Then, one can determine the vote of the *j*th sensing node for the *m*th modulation candidate $M_{m}\in M$ as

(6) $$V_{m,j,k}\overset{\mathrm{def}}{=}\frac{1}{{\hat{H}}_{m,j,k}},\;\;\mathrm{where}\;{\hat{H}}_{m,j,k}\in {\hat{H}}_{j,k}.$$

Thus, in the *k*th sensing interval, the overall vote for $M_{m}\in M$ over all sensing nodes can be calculated as

(7) $${\tilde{V}}_{m,k}\overset{\mathrm{def}}{=}\sum_{j=1}^{J}V_{m,j,k},$$

and the collection of votes $V_{k}\overset{\mathrm{def}}{=}\left\{{\tilde{V}}_{1,k},{\tilde{V}}_{2,k},\cdots ,{\tilde{V}}_{M,k}\right\}$ corresponding to the entire modulation candidate set M can be subsequently obtained. Consequently, the global decision on the modulation type in the *k*th sensing interval can be made by picking the modulation candidate in M with the maximum vote as expressed by

(8) $$D_{k}=\underset{M_{m}\in M}{\mathrm{argmax}}\;{\tilde{V}}_{m,k},$$

It should be pointed out that, if multiple modulation schemes in M obtain the same highest vote in a certain sensing interval, any of them can be picked randomly as the global decision.

In summary, the details of our proposed new CAMC approach using vectorized soft decision fusion for WSNs can be manifested by Algorithm 1 below.
**Algorithm 1:** Our proposed new CAMC scheme using vectorized soft decision fusion for WSNs.**Input:** 
a sensing interval index *k*, the signal sequences $x_{j,k}\left(n\right)$ received by the *j*th sensing node in the *k*th sensing interval, j=1, 2, *…*, J, the number of sensing nodes within the WSN in the *k*th sensing interval, the preset flooring constant ζ and the modulation candidate set $M\overset{\mathrm{def}}{=}\left\{M_{1},M_{2},\cdots ,M_{M}\right\}$.**Output:** 
the global decision $D_{k}$ for the *k*th sensing interval.
1:In the *k*th sensing interval, generate the local NHD sequences $H_{j,k}$, j=1, 2, *…*, J, for all modulation candidates in M, according to the graph-based AMC technique proposed in [22];2:Convert $H_{j,k}$ to the “perturbed” local NHD sequence ${\hat{H}}_{j,k}$, for j=1, 2, *…*, J, according to Equation (5);3:Determine the vote of the *j*th sensing node for the *m*th modulation candidate $M_{m}\in M$ based on the corresponding element of ${\hat{H}}_{j,k}$ using Equation (6);4:Calculate the overall vote for $M_{m}\in M$ over all sensing nodes in the *k*th sensing interval, ${\tilde{V}}_{m,k}$, according to Equation (7);5:Collect all votes ${\tilde{V}}_{m,k}$, for m=1, 2, *…*, *M* to form the set of votes $V_{k}\overset{\mathrm{def}}{=}\left\{{\tilde{V}}_{1,k},{\tilde{V}}_{2,k},\cdots ,{\tilde{V}}_{M,k}\right\}$ for all modulation candidates in M;6:Make the global decision $D_{k}$ based on the set of votes $V_{k}$ corresponding to the entire modulation candidate set M according to Equation (8);7:**return**$D_{k}$

### 3.3. Computational Complexity Analysis

The computational complexity of our proposed new CAMC approach using vectorized soft decision fusion for WSNs is theoretically investigated here. According to the framework of our proposed CAMC approach, its computational complexity involves three parts, including the complexities required for the local graph-based AMC, the vote generation and the soft decision fusion based on voting. During the *k*th sensing interval, the graph-based AMC technique is invoked by each sensing node to generate the set of NHDs. According to [22], the computational complexity of the single-node graph-based AMC method is $O(N^{2})$ arithmetic operations, where *N* denotes the sample size of the received signal at each local sensing node. Then, the sets of NHDs generated by local sensing nodes are conveyed to the FC and the votes corresponding to M are subsequently calculated, which involves O(1) arithmetic operations. The global decision is made at the FC by voting, where the computational complexity of this soft decision fusion is O(1). Thus, the overall computational complexity of our proposed new CAMC approach using vectorized soft decision fusion (i.e., VSDF CAMC scheme) for WSNs is $O\left(N^{2}\right)+O\left(1\right)$.

On the other hand, the computational complexities of the exiting CAMC method using the credit-based consensus fusion rule presented in [23] and the optimal hard-decision fusion (OHDF) CAMC approach proposed in [19] are also estimated for comparison. For the exiting credit-based CAMC method in [23], its computational complexity can be directly divided into three parts, including the complexity required for the AMC based on local graphs, the local decision making and the ultimate decision fusion based on weighted voting. The corresponding computational complexities of these three parts are $O(N^{2})$, O(1) and O(1), respectively. The overall computational complexity of the credit-based CAMC approach in [23] is $O\left(N^{2}\right)+O\left(1\right)$. Meanwhile, the OHDF CAMC approach also consists of three parts, including the complexities required for the local graph-based AMC, the TFC (tentative fusion center) selection and the decision fusion based on weighted voting. The corresponding computational complexities of these three parts are $O(N^{2})$, O(N) and O(1), respectively. The overall computational complexity of the OHDF CAMC approach in [19] is thus $O\left(N^{2}\right)+O\left(N\right)+O\left(1\right)$. Finally, the computational complexities of our proposed new VSDF CAMC approach, the existing credit-based CAMC method and the existing OHDF CAMC method are compared by Table 1. It is conspicuous that our proposed new VSDF CAMC approach possesses the same overall computational complexity as the exiting credit-based CAMC method proposed in [23] and can effectively reduce the computational complexity in comparison with the existing OHDF CAMC technique proposed in [19].

### 3.4. Transmission-Overhead Analysis

The transmission overheads required by our proposed new VSDF CAMC approach and the two existing methods presented in [19,23] were investigated under a WSN containing the same number of sensing nodes. Let us assume that the WSN consists of J sensing nodes and the number of modulation candidates in M is *M*. In the *k*th sensing interval, according to our proposed new VSDF CAMC method, all sensing nodes transmit their local NHD sequences corresponding to all of the modulation candidates in M to a separate FC and the global CAMC decision is made by the weighted vote which results from all sensing nodes’ NHD sequences. The total number of the required transmissions for global decision making is thus J×M. Meanwhile, the existing credit-based CAMC method proposed in [23] undertakes CAMC at a separate FC based on the local decisions generated by all of the sensing nodes within the WSN. Since each sensing node transmits its own decision to the FC only once during each sensing interval, the total number of the required transmissions for decision fusion is J. On the other hand, the existing OHDF CAMC method proposed in [19] dynamically selects a sensing node in the WSN as a tentative fusion center (TFC) to make the global decision according to the local identification decisions transmitted by other sensor nodes. The total number of transmissions in [19] is J−1. In summary, the total transmission overheads of our proposed new VSDF CAMC approach and its counterparts are listed in Table 2. According to Table 2, the transmission overhead resulting from our proposed new VSDF CAMC approach is higher than the two existing counterparts. However, such extra cost in transmission overhead is worthwhile for the classification performance improvement.

## 4. Numerical Simulation and Comparative Study

In this section, we present the results of our proposed new CAMC approach using the vectorized soft decision fusion rule evaluated via Monte Carlo simulations, in terms of *correct classification probability* $P_{cc}$ versus average signal-to-noise ratio (SNR) η over all sensing nodes (since the noise power at each node may be different from another). Generally speaking, there are *M* modulation candidates for classification, which are represented by the set $\left\{M_{1},M_{2},\cdots ,M_{M}\right\}$. Thus, $P_{cc}$ can be formulated as

(9) $$P_{\mathrm{cc}}\overset{\mathrm{def}}{=}\sum_{m=1}^{M}P\left(M_{m}|M_{m}\right)\;P_{M_{m}},$$

where $P_{M_{m}}$ denotes the probability of the modulation $M_{m}$ occurrence and $P\left(M_{m}|M_{m}\right)$ represents the correct classification probability when the modulation $M_{m}$ is transmitted.

Our method was also compared with the existing CAMC technique (we did not compare the existing soft-decision-based CAMC method in [20], because the fourth-order cumulant method therein cannot classify constant-modulus signals) using the credit-based consensus fusion rule proposed in [23] and the existing OHDF CAMC method in [19] and the advantage of our proposed new CAMC method was also theoretically studied using the linear discriminant analysis (LDA). Here, the modulation candidate set M employed in Monte Carlo simulations included six common modulation types, namely, BPSK (binary phase-shift keying), 2FSK (binary frequency-shift keying), 4FSK (quadrature frequency-shift keying), QPSK (quadrature phase-shift keying), OQPSK (offset quadrature phase-shift keying) and MSK (minimum shift keying); consequently, M=6. A wireless sensing network with centralized architecture consisted of a fusion center (FC) in tandem with nine sensing-devices and there were different multipath Rayleigh fading channels between the target signal and the nine sensing nodes (J=9). These fading channels were characterized as shown in Table 3. In each trial of the Monte Carlo simulation, an unknown target signal s(n) with a modulation type defined in the preset modulation candidate set M and the noise power arising from the propagation channel were randomly generated by the computer, where the sample size of s(n) was chosen to be 10,000 and the SNR of the individual received signal could be randomly set in the range of $\left[-20\;\mathrm{dB},20\;\mathrm{dB}\right]$. For the graph-based AMC method at local sensing nodes, the FFT window size of the composite demodulation in FAM was set to 32. For feature extraction of the received signals with different modulations, we adopted the NHD sequence as the feature vector for each modulation of the candidate set M and the length of the NHD sequence (feature vector) was L=1; the total number of features generated by all sensing nodes considered here was L×M×J=54. For the same system set-up, a thousand Monte Carlo trials were performed to obtain the average AMC accuracies with respect to different average SNRs.

### 4.1. Effectiveness of Our Proposed CAMC Method

**Figure 2:**
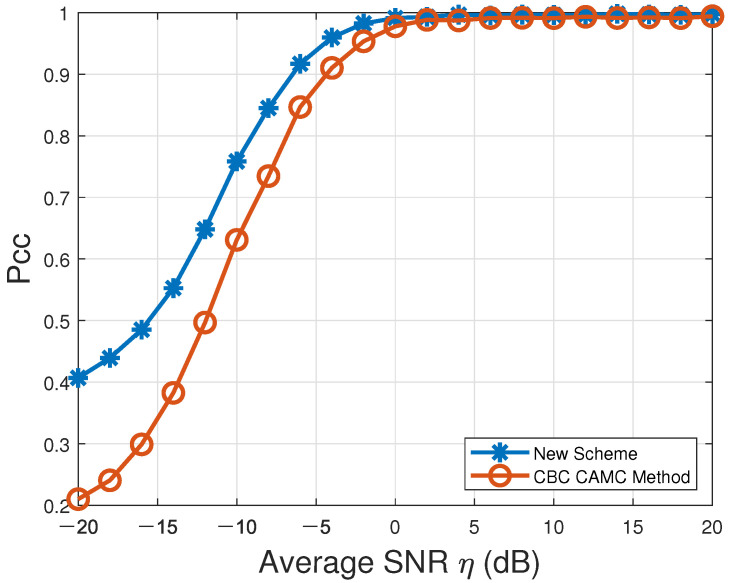
Probabilities of correct classification, $P_{\mathrm{cc}}$, versus average SNR for CAMC over the modulation candidate set $M\overset{\mathrm{def}}{=}\left\{\mathrm{BPSK},\mathrm{OQPSK},\mathrm{QPSK},2\mathrm{FSK},4\mathrm{FSK},\mathrm{MSK}\right\}$.

Our proposed novel CAMC scheme was compared with the aforementioned CAMC method using credit-based consensus fusion proposed in [23]. Here, the network topology and other simulation parameters remained the same as those adopted in [23], which are also listed in Table 4. For comparing our proposed new VSDF CAMC scheme and the existing CAMC method using credit-based consensus fusion, the respective probabilities of correct classification $P_{\mathrm{cc}}$ over the entire modulation candidate set M in the presence of Rayleigh multipath channels characterized by Table 3 and AWGN are listed in Table 4 and depicted in Figure 2. According to Figure 2, these two CAMC methods could both reach up to $P_{\mathrm{cc}}\approx 100\%$ (perfect AMC accuracy) when η≥2dB. Our proposed new CAMC approach (denoted by “New Scheme” in the figure) could lead to $P_{\mathrm{cc}}=76\%$ when η≥−10dB. However, in order to achieve the same accuracy, the existing CAMC method using credit-based consensus fusion (denoted by “CBC CAMC Method” in the figure) required the average SNR to be at least −7dB. It is obvious that our proposed new CAMC scheme remarkably outperformed the existing decision-level CAMC method proposed in [23], especially for low average SNRs.

**Figure 3:**
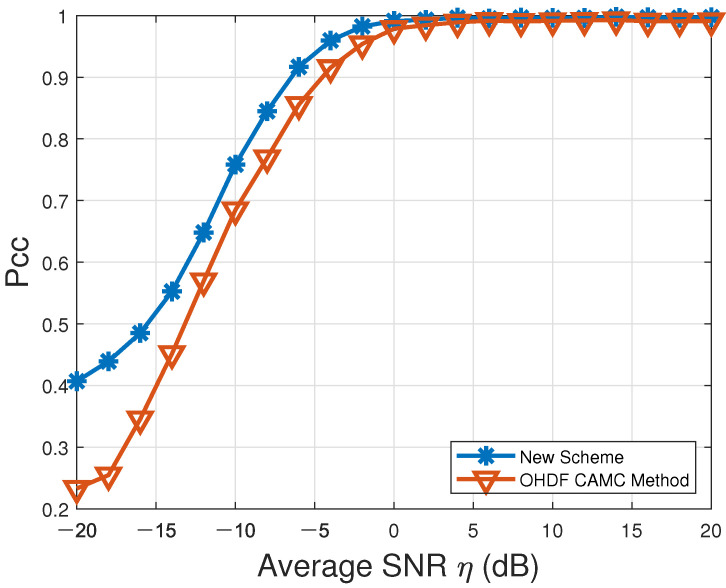
Probabilities of correct classification, $P_{\mathrm{cc}}$, versus average SNR for CAMC over the modulation candidate set $M\overset{\mathrm{def}}{=}\left\{\mathrm{BPSK},\mathrm{OQPSK},\mathrm{QPSK},2\mathrm{FSK},4\mathrm{FSK},\mathrm{MSK}\right\}$.

Meanwhile, our proposed new VSDF CAMC scheme was also compared with the existing OHDF CAMC method proposed in [19], since our proposed new VSDF CAMC approach can be directly employed to undertake CAMC by a WSN. Here, the modulation candidate set and the simulation conditions remained the same as those adopted in [19]. The respective $P_{\mathrm{cc}}$ values over the entire modulation candidate set M in the presence of Rayleigh multipath channels characterized by Table 3 and AWGN are depicted in Figure 3 and listed in Table 5. According to Figure 3, the $P_{\mathrm{cc}}$ values of our proposed new VSDF CAMC approach and the existing OHDF CAMC method proposed in [19] (denoted by “New Scheme” and “OHDF CAMC Method” in the figure, respectively) could both reach up close to 100% (perfect AMC accuracy) when η≥2dB. However, the $P_{\mathrm{cc}}$ resulting from our proposed new VSDF CAMC approach was significantly higher than that produced by the existing OHDF CAMC method in low average SNRs (η≤−5dB). Thus, the recognition accuracy achieved by our proposed new VSDF CAMC scheme was superior to that resulting from the existing OHDF CAMC method in [19], especially for low average SNRs.

### 4.2. Performance Comparison between Our Proposed New CAMC Scheme and the Existing Single-Node AMC Methods

To demonstrate the superiority of our proposed new VSDF CAMC approach to the existing single-node AMC methods in terms of classification accuracy, our proposed novel CAMC approach using the vectorized soft decision fusion rule was compared with the existing single-node graph-based AMC method in [22] and the existing single-node AMC scheme based on high-order statistics (HOS) in [24].

**Figure 4:**
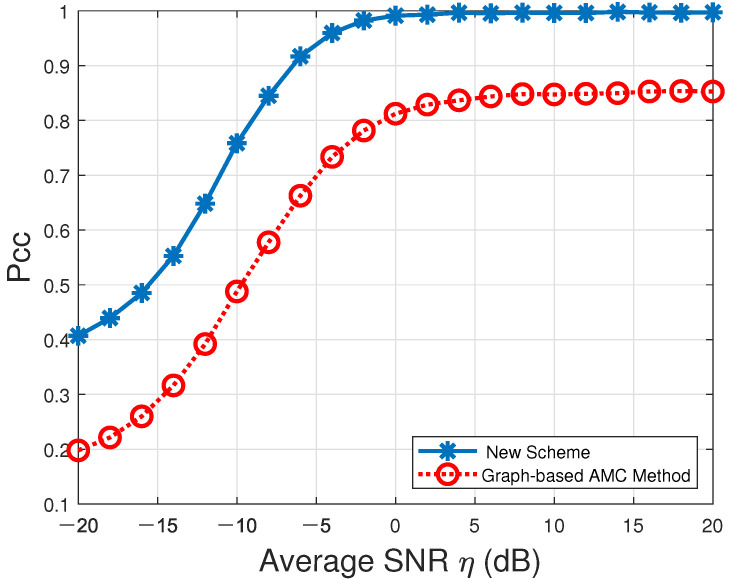
Probabilities of correct classification, $P_{\mathrm{cc}}$, versus average SNR for our proposed new VSDF CAMC approach and the existing single-node graph-based AMC method in [22] in the presence of multipath Rayleigh channels and AWGN over the modulation candidate set $M\overset{\mathrm{def}}{=}\left\{\mathrm{BPSK},\mathrm{OQPSK},\mathrm{QPSK},2\mathrm{FSK},4\mathrm{FSK},\mathrm{MSK}\right\}$.

The probabilities of correct classification $P_{\mathrm{cc}}$ over the entire modulation candidate set M in the presence of Rayleigh multipath channels characterized by Table 3 and AWGN are listed in Table 6 and depicted in Figure 4.

According to Figure 4, the $P_{\mathrm{cc}}$ values produced by our proposed new VSDF CAMC approach (denoted by “New Scheme” in the figure) could always converge to 100% when η≥0dB. On the contrary, the $P_{\mathrm{cc}}$ values resulting from the single-node graph-based AMC method (denoted by “Graph-based AMC Method”in the figure) could not reach up to 100% across the entire average SNR range. Thus, our proposed new CAMC approach could significantly improve the individual local AMC accuracy.

**Figure 5:**
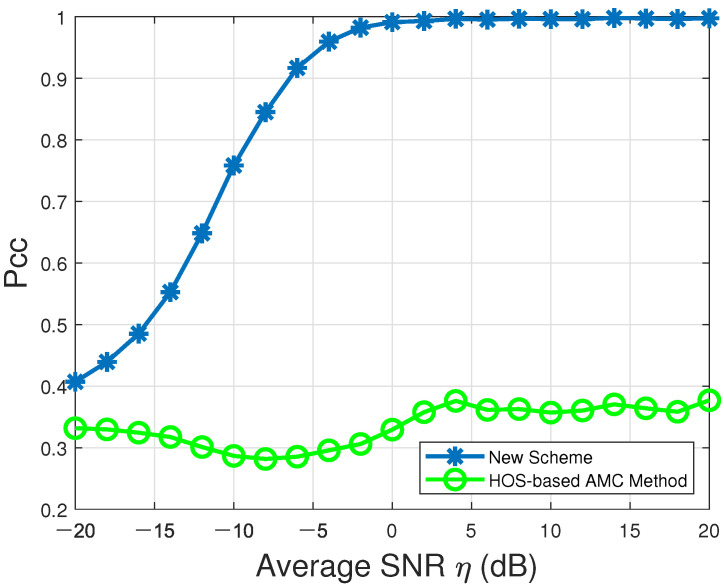
Probabilities of correct classification, $P_{\mathrm{cc}}$, versus average SNR for our proposed new VSDF CAMC approach and the existing single-node HOS-based AMC method in [24] in the presence of multipath Rayleigh channels and AWGN over the modulation candidate set $\tilde{M}\overset{\mathrm{def}}{=}\left\{\mathrm{BPSK},2\mathrm{FSK},4\mathrm{FSK}\right\}$.

Meanwhile, the existing single-node HOS-based AMC method in [24] was also compared with our proposed new CAMC scheme here. According to [24], the HOS-based AMC method has to utilize a huge number of signal samples to reliably estimate the HOS parameters of the received signal, including $c_{40}$, $c_{42}$ and $c_{21}^{2}$, which are adopted to facilitate the modulation features $f_{1}$ and $f_{5}$ for AMC. Refer to [24] for details. Since the modulation candidate set for the existing HOS-based AMC method in [24] can only contain three modulation types, namely, BPSK, 2FSK and 4FSK, we had to reduce the modulation candidate set $\tilde{M}$ to include these three types only for fair comparison, such that $\tilde{M}\overset{\mathrm{def}}{=}\left\{{\tilde{M}}_{1},{\tilde{M}}_{2},{\tilde{M}}_{3}\right\}$. The probabilities of correct classification $P_{\mathrm{cc}}$ for the entire modula- tion candidate set $\tilde{M}$ in the presence of Rayleigh multipath channels specified by Table 3 and AWGN are listed in Table 7 and delineated by Figure 5. According to Figure 5, the existing single-node HOS-based AMC method (denoted by “HOS-based AMC Method” in the figure) could not lead to any promising result, even in high average SNR conditions. On the other hand, our proposed new VSDF CAMC scheme (denoted by “New Scheme” in the figure) could reach up to 100% when the average SNR was as low as −6dB.

Thus, our proposed new VSDF CAMC scheme remarkably outperformed the existing single-node graph-based and HOS-based AMC methods.

### 4.3. Comparative Study between Our Proposed New CAMC Scheme and the Existing CAMC Method

**Figure 6:**
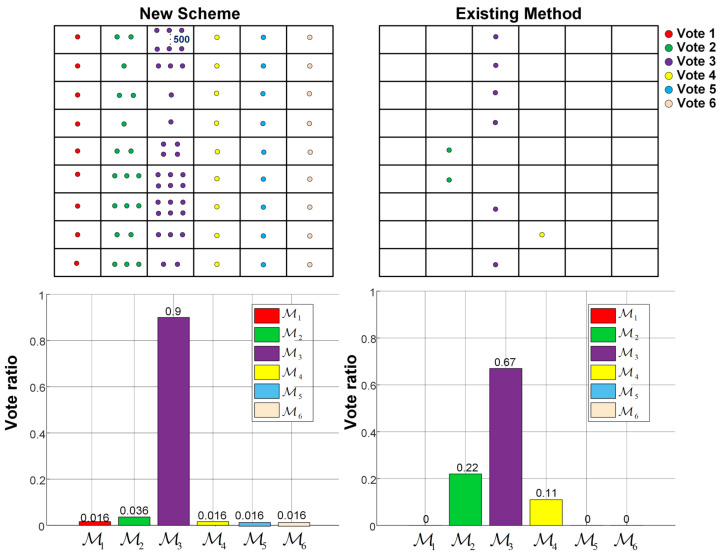
Illustration of different voting results from our proposed new CAMC scheme and the existing CAMC method in [23] for a certain sensing interval.

Since the global decisions resulting from our proposed new CAMC approach using the vectorized soft decision fusion rule and the existing decision-level CAMC technique using credit-based consensus fusion in [23] are both based on voting, the discrepancy between the corresponding voting mechanisms to these two approaches in a certain sensing interval is illustrated by Figure 6. Here, the topology of WSN and the modulation candidate set M were retained. At the top of Figure 6, the nine sensing nodes and their votes for the six modulation candidates resulting from the two aforementioned approaches (denoted by “New Scheme” and “Existing Method”, respectively) in the *k*th sensing interval are shown by a 9×6 matrix, where the number of dots in the cell at row *j* and column *m*, j=1, 2, *…*, 9, m=1, 2, *…*, 6 denotes the vote contributed by the *j*th sensing node for the *m*th modulation candidate $M_{m}$. Besides, the bar plots at the bottom of Figure 6 demonstrate the corresponding vote ratios (the total vote for a modulation candidate over all sensing nodes divided by the sum of the total votes over all candidates) for the modulation candidates in M to the two aforementioned methods. According to Figure 6, although the total votes for any modulation candidate resulting from these two approaches are different, two CAMC methods may still reach the same global decision ($M_{3}$, as illustrated by Figure 6) corresponding to the maximum vote ratio. By use of our proposed new CAMC method, the maximum vote ratio corresponding to the modulation candidate $M_{3}$ turned out to be 0.9, while the second largest vote ratio corresponding to $M_{2}$ was 0.036. Using our proposed new CAMC scheme, the difference in the vote ratios between the identified modulation candidate and the runner-up candidate is as large as 0.864. On the other hand, such difference resulting from the existing method in [23] is only 0.45. Thus, our proposed new CAMC approach could lead to more reliable voting results for robust global decision making than the existing CAMC method in [23] without the vectorized soft decision fusion mechanism.

Furthermore, we employed the multi-class linear discriminant analysis (LDA) in [25] to compare the different voting results across twenty-one consecutive sensing intervals resulting from the two aforementioned CAMC methods. In the *k*th sensing interval, one can collect all votes $V_{m,j,k}$, m=1, 2, *…*, *M* and j=1, 2, *…*, J at the FC and ${\bar{V}}_{m,k}\overset{\mathrm{def}}{=}[V_{m,1,k},V_{m,2,k},\cdots ,$$V_{m,J,k}{]}^{T}$ denotes the *vote vector* corresponding to the modulation candidate $M_{m}\in M$. Then, the intra-class divergence matrix $S_{w}\left(k\right)$ and inter-class divergence matrix $S_{b}\left(k\right)$ can be obtained as

(10) $$S_{w}\left(k\right)\overset{\mathrm{def}}{=}\sum_{m=1}^{M}\left({\bar{V}}_{m,k}-\mu_{m,k}\;\bar{1}\right){\left({\bar{V}}_{m,k}-\mu_{m,k}\;\bar{1}\right)}^{T},$$

(11) $$S_{b}\left(k\right)\overset{\mathrm{def}}{=}\sum_{m=1}^{M}\left({\bar{U}}_{k}-\mu_{m,k}\;\bar{1}\right){\left({\bar{U}}_{k}-\mu_{m,k}\;\bar{1}\right)}^{T},$$

where

(12) $$\begin{array}{c} \mu_{m,k}\overset{\mathrm{def}}{=}\frac{1}{J}\sum_{j=1}^{J}V_{m,j,k}, \end{array}$$

represents the average vote for the modulation candidate $M_{m}\in M$ in the *k*th sensing interval,

(13) $${\bar{U}}_{k}\overset{\mathrm{def}}{=}\frac{1}{M}\sum_{m=1}^{M}{\bar{V}}_{m,k},$$

represents the average-vote vector over the entire modulation candidate set M in the *k*th sensing interval and $\bar{1}$ denotes the J×1 all-one vector. Note that $S_{w}\left(k\right)$ is usually considered a non-singular matrix in LDA. However, $S_{w}\left(k\right)$ is not necessarily a full-rank matrix in practice, which should be replaced by the total scatter matrix $S_{t}\left(k\right)$ as given by

(14) $$S_{t}\left(k\right)\overset{\mathrm{def}}{=}S_{w}\left(k\right)+S_{b}\left(k\right).$$

**Figure 7:**
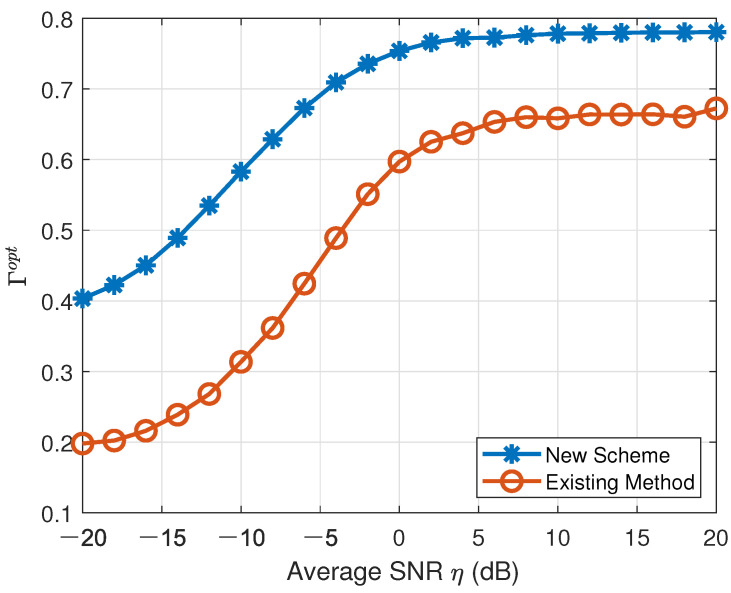
Comparison of $\Gamma^{opt}$ resulting from our proposed new CAMC scheme and the existing CAMC method in [23] based on LDA across twenty-one consecutive sensing intervals.

Consequently, the objective for LDA is given by

(15) $$W^{\mathrm{opt}}\overset{\mathrm{def}}{=}\underset{W}{\mathrm{argmax}}\;\frac{W^{T}S_{b}\left(k\right)\;W}{W^{T}S_{t}\left(k\right)\;W},$$

where *W* is a 1×J optimal projection vector according to [26]. We define

(16) $$\begin{array}{c} \Gamma^{\mathrm{opt}}\overset{\mathrm{def}}{=}\underset{W}{\max}\;\frac{W^{T}S_{b}\left(k\right)\;W}{W^{T}S_{t}\left(k\right)\;W}, \end{array}$$

where $\Gamma^{\mathrm{opt}}$ reflects the average normalized inter-class distance (discrepancy) over the total votes for individual modulation candidates. The larger $\Gamma^{\mathrm{opt}}$ a CAMC scheme results in, the better distinguishability it possesses. According to the total votes produced by our proposed new CAMC approach and the existing CAMC method in [23], the corresponding metrics $\Gamma^{\mathrm{opt}}$ were computed versus the average SNR and are depicted in Figure 7. According to Figure 7, the metric $\Gamma^{\mathrm{opt}}$ resulting from our proposed new CAMC method (denoted by “New Scheme” in the figure) was always significantly larger than that resulting from the existing CAMC method (denoted by “Existing Method” in the figure) in [23], which means that our proposed new CAMC scheme had much better modulation distinguishability than the existing CAMC scheme in [23]. The advantage of our proposed new CAMC scheme as shown by Figure 2 is thus manifested by Figure 7 and Table 8.

## 5. Conclusions

In this paper, we propose a new cooperative automatic modulation classification (CAMC) method using the vectorized soft decision fusion rule. At the training stage, a training graph-feature sequence is generated from each modulation candidate. During the test, in a sensing interval, each local sensing node first produces the test graph-feature sequence corresponding to each modulation candidate. The normalized Hamming distances between the training and test graph-feature sequences corresponding to all modulation candidates are collected and transmitted to the fusion center. Finally, the fusion center makes the global decision based on the new vectorized soft decision fusion rule. According to Monte Carlo simulations, our proposed new CAMC scheme could lead to $P_{\mathrm{cc}}=76\%$, even when η≥−10dB, and reach up to $P_{\mathrm{cc}}\approx 100\%$, when η≥0dB. Based on the linear discriminant analysis, the average classification accuracy of our proposed new CAMC method was higher than that of the existing CAMC method using the credit-based consensus fusion rule, especially for low signal-to-noise ratios.

## Figures and Tables

**Table 1:** Computational complexities of our proposed new VSDF CAMC approach, the existing credit-based CAMC method in [23] and the existing OHDF CAMC method in [19].

Method	Constituents	Computational Complexity	Overall Computational Complexity
New VSDF CAMC	Local graph-based AMC	$O(N^{2})$	$O\left(N^{2}\right)+O\left(1\right)$
	Individual vote generation	O(1)	
	Soft decision fusion	O(1)	
Credit-based CAMC	Local graph-based AMC	$O(N^{2})$	$O\left(N^{2}\right)+O\left(1\right)$
	Local decision making	O(1)	
	Decision fusion	O(1)	
OHDF CAMC	Local graph-based AMC	$O(N^{2})$	$O\left(N^{2}\right)+O\left(N\right)+O\left(1\right)$
	TFC selection	O(N)	
	Decision fusion	O(1)	

**Table 2:** The numbers of required transmissions of our proposed VSDF CAMC approach, the existing credit-based CAMC Method in [23], and the existing OHDFCAMC method in [19] during one sensing interval.

	New VSDF CAMC	Credit-Based CAMC	OHDF CAMC
Number of Sensing Nodes	J	J	J
Number of Modulation Candidates	*M*	*M*	*M*
Number of Transmissions	JM	J	J−1

**Table 3:** Delay and power profiles for multipath Rayleigh fading channels for individual sensing nodes.

Parameters	Path Time Delays (ms)	Path Power Profile (dB)
Channel 1	[0.2,2,4]	[0,−2,−6]
Channel 2	[0.4,0.6,8]	[−2,−4,−6]
Channel 3	[0.04,0.2,8]	[−2,−4,−10]
Channel 4	[0.08,0.4,0.2,1]	[0,−2,−4,−8]
Channel 5	[0.04,0.08,4]	[−2,−4,−10]
Channel 6	[0.01,0.3,6]	[0,−8,−16]
Channel 7	[0.2,6,8]	[0,−10,−20]
Channel 8	[0.02,0.4,0.8,6]	[−2,−6,−10,−16]
Channel 9	[0.06,0.8,2]	[−4,−6,−12]

**Table 4:** The parameter setting and the $P_{\mathrm{cc}}$’s of our proposed CAMC approach and the existing CBC CAMC method in [23].

**Parameter Setting**	Number of Sensing Nodes J		9
	Modulation Candidate Set M		BPSK, OQPSK, QPSK, 2FSK, 4FSK, MSK
	Flooring Constant ζ		$10^{-5}$
	FFT Window Size in FAM		32
	Sample Size		10,000
	Number of Monte Carlo Trials		1000
	Average SNR Range		[−20 dB:2 dB:20 dB]
**Simulation Results**	**Average SNR**	$P_{\mathrm{cc}}$ **for the Proposed CAMC Method**	$P_{\mathrm{cc}}$ **for the Existing CBC CAMC Method**
	−20dB	0.4070	0.2332
	−18dB	0.4393	0.2548
	−16dB	0.4852	0.3457
	−14dB	0.5527	0.4517
	−12dB	0.6482	0.5697
	−10dB	0.7583	0.6848
	−8dB	0.8450	0.7692
	−6dB	0.9168	0.8557
	−4dB	0.9595	0.9155
	−2dB	0.9823	0.9540
	0dB	0.9912	0.9787
	2dB	0.9930	0.9843
	4dB	0.9965	0.9890
	6dB	0.9953	0.9917
	8dB	0.9967	0.9903
	10dB	0.9962	0.9915
	12dB	0.9960	0.9920
	14dB	0.9980	0.9928
	16dB	0.9970	0.9907
	18dB	0.9963	0.9907
	20dB	0.9973	0.9912

**Table 5:** The parameter setting and the $P_{\mathrm{cc}}$’s of our proposed CAMC Approach and the existing OHDF CAMC method in [19].

**Parameter Setting**	Number of Sensing Nodes J		9
	Modulation Candidate Set M		BPSK, OQPSK, QPSK, 2FSK, 4FSK, MSK
	FFT Window Size in FAM		32
	Sample Size		10,000
	Number of Monte Carlo Trials		1000
	Average SNR Range		[−20 dB:2 dB:20 dB]
**Simulation Results**	**Average SNR**	$P_{\mathrm{cc}}$ **for the Proposed CAMC Method**	$P_{\mathrm{cc}}$ **for the Existing OHDF CAMC Method**
	−20dB	0.4070	0.2332
	−18dB	0.4393	0.2548
	−16dB	0.4852	0.3457
	−14dB	0.5527	0.4517
	−12dB	0.6482	0.5697
	−10dB	0.7583	0.6848
	−8dB	0.8450	0.7692
	−6dB	0.9168	0.8557
	−4dB	0.9595	0.9155
	−2dB	0.9823	0.9540
	0dB	0.9912	0.9787
	2dB	0.9930	0.9843
	4dB	0.9965	0.9890
	6dB	0.9953	0.9917
	8dB	0.9967	0.9903
	10dB	0.9962	0.9915
	12dB	0.9960	0.9920
	14dB	0.9980	0.9928
	16dB	0.9970	0.9907
	18dB	0.9963	0.9907
	20dB	0.9973	0.9912

**Table 6:** The parameter setting and the $P_{\mathrm{cc}}$’s of our proposed CAMC approach and the existing single-node graph-based AMC method in [22].

**Parameter Setting**	**Parameter**	**The Proposed CAMC Method**	**Single-Node Graph-Based AMC**
	Number of Sensing Nodes J	9	1
	Modulation Candidate Set M	BPSK, OQPSK, QPSK, 2FSK, 4FSK, MSK	BPSK, OQPSK, QPSK, 2FSK, 4FSK, MSK
	Flooring Constant ζ	$10^{-5}$	-
	FFT Window Size in FAM	32	32
	Sample Size	10,000	10,000
	Number of Monte Carlo Trails	1000	1000
	Average SNR Range	[−20 dB:2 dB:20 dB]	[−20 dB:2 dB:20 dB]
**Simulation Results**	**Average SNR**	$P_{\mathrm{cc}}$ **for the Proposed CAMC Method**	$P_{\mathrm{cc}}$ **for Single-Node Graph-Based AMC**
	−20dB	0.4070	0.1979
	−18dB	0.4393	0.2215
	−16dB	0.4852	0.2600
	−14dB	0.5527	0.3164
	−12dB	0.6482	0.3920
	−10dB	0.7583	0.4879
	−8dB	0.8450	0.5776
	−6dB	0.9168	0.6626
	−4dB	0.9595	0.7335
	−2dB	0.9823	0.7816
	0dB	0.9912	0.8123
	2dB	0.9930	0.8828
	4dB	0.9965	0.8367
	6dB	0.9953	0.8437
	8dB	0.9967	0.8481
	10dB	0.9962	0.8474
	12dB	0.9960	0.8487
	14dB	0.9980	0.8497
	16dB	0.9970	0.8528
	18dB	0.9963	0.8537
	20dB	0.9973	0.8528

**Table 7:** The parameter setting and the $P_{\mathrm{cc}}$’s of our proposed CAMC approach and the existing HOS-based AMC method in [24].

**Parameter Setting**	**Parameter**	**The Proposed CAMC Method**	**Single-Node HOS-Based AMC**
	Number of Sensing Nodes J	9	1
	Modulation Candidate Set M	BPSK, 2FSK, 4FSK	BPSK, 2FSK, 4FSK
	Flooring Constant ζ	$10^{-5}$	-
	FFT Window Size in FAM	32	32
	Sample Size	10,000	12,000
	Number of Monte Carlo Trails	1000	1000
	Average SNR Range	[−20 dB:2 dB:20 dB]	[−20 dB:2 dB:20 dB]
**Simulation Results**	**Average SNR**	$P_{\mathrm{cc}}$ **for the Proposed CAMC Method**	$P_{\mathrm{cc}}$ **for Single-Node HOS-Based AMC**
	−20dB	0.4070	0.3317
	−18dB	0.4393	0.3297
	−16dB	0.4852	0.3243
	−14dB	0.5527	0.3173
	−12dB	0.6482	0.3010
	−10dB	0.7583	0.2867
	−8dB	0.8450	0.2817
	−6dB	0.9168	0.2853
	−4dB	0.9595	0.2957
	−2dB	0.9823	0.3060
	0dB	0.9912	0.3293
	2dB	0.9930	0.3577
	4dB	0.9965	0.3760
	6dB	0.9953	0.3613
	8dB	0.9967	0.3627
	10dB	0.9962	0.3570
	12dB	0.9960	0.3603
	14dB	0.9980	0.3703
	16dB	0.9970	0.3640
	18dB	0.9963	0.3583
	20dB	0.9973	0.3770

**Table 8:** The parameter setting and $\Gamma^{opt}$ resulting from our proposed CAMC approach and the existing CAMC method in [23].

**Parameter Setting**	Number of Sensing Nodes J		9
	Modulation Candidate Set M		BPSK, OQPSK, QPSK, 2FSK, 4FSK, MSK
	Flooring Constant ζ		$10^{-5}$
	FFT Window Size in FAM		32
	Sample Size		10,000
	Number of Monte Carlo Trails		1000
	Average SNR Range		$\left[-20\;\mathrm{dB}:2\;\mathrm{dB}:20\;\mathrm{dB}\right]$
**Simulation Results**	**Average SNR**	$\Gamma^{\mathrm{opt}}$ **for the Proposed CAMC Method**	$\Gamma^{\mathrm{opt}}$ **for the Existing CAMC Method**
	−20 dB	0.4033	0.1981
	−18 dB	0.4224	0.2024
	−16 dB	0.4503	0.2161
	−14 dB	0.4891	0.2389
	−12 dB	0.5348	0.2683
	−10 dB	0.5829	0.3137
	−8 dB	0.6288	0.3617
	−6 dB	0.6729	0.4244
	−4 dB	0.7093	0.4889
	−2 dB	0.7355	0.5510
	0 dB	0.7538	0.5971
	2 dB	0.7657	0.6252
	4 dB	0.7718	0.6374
	6 dB	0.7726	0.6534
	8 dB	0.7761	0.6600
	10 dB	0.7782	0.6584
	12 dB	0.7787	0.6638
	14 dB	0.7794	0.6637
	16 dB	0.7799	0.6641
	18 dB	0.7800	0.6606
	20 dB	0.7804	0.6724
